# Partner notification outcomes after integration of an on-site disease intervention specialist at a sexually transmitted disease clinic

**DOI:** 10.1371/journal.pone.0194041

**Published:** 2018-03-27

**Authors:** Alec Tributino, Madeline C. Montgomery, Thomas Bertrand, Theodore Marak, Alexi Almonte, Jacob van den Berg, Kristen St. John, Carol Browning, Martha M. Medina, Ashley Morse, Philip A. Chan

**Affiliations:** 1 Department of Medicine, Brown University, Providence, Rhode Island, United States of America; 2 Center for HIV, Hepatitis, Sexually Transmitted Diseases, and Tuberculosis Epidemiology, Rhode Island Department of Health, Providence, Rhode Island, United States of America; 3 Department of Behavioral and Social Sciences, Brown University School of Public Health, Providence, Rhode Island, United States of America; Massachusetts General Hospital, UNITED STATES

## Abstract

**Background:**

Partner notification services (PNS) are highly effective in reducing transmission of sexually transmitted diseases (STDs). We assessed outcomes of PNS before and after integration of an on-site disease intervention specialist (DIS) at a publicly-funded STD clinic.

**Methods:**

From August 2014 to December 2015, patients testing positive for infectious syphilis or gonorrhea at the Rhode Island STD Clinic were referred to on-site DIS for partner notification. Data on PNS outcomes were reviewed for eight months before integration of DIS at the clinic and compared to eight months after.

**Results:**

Of the 145 index patients referred for PNS during the study period (n = 58 before DIS integration, n = 87 after), 86% were interviewed. DIS integration resulted in a significantly greater proportion of index patients interviewed overall (92% versus 76%, p<0.01), on the day of diagnosis (85% versus 61%; p<0.01), and in person at the STD clinic (64% versus 11%; p<0.01). However, there was no significant difference in number of sexual partners named or treated.

**Conclusions:**

Integrating DIS at a publicly-funded STD clinic resulted in a greater number of index cases interviewed, a greater number interviewed in person, and a greater number interviewed on the day of diagnosis. Challenges remain in identifying and engaging partners for treatment.

## Introduction

Sexually transmitted diseases (STDs) are a public health crisis in the United States (US), with the prevalence of chlamydia, gonorrhea, and syphilis increasing dramatically over the last ten years [1]. In 2016, the total number of cases of gonorrhea and infectious syphilis increased by 19% and 17%, respectively, compared to the prior year. Young people aged 15–24 years, people of color, and gay, bisexual, and other men who have sex with men (MSM) experience a particularly high burden of disease [1]. In 2016, there was a disproportionate number of gonorrhea cases (51%) diagnosed among Black individuals and MSM. Young people also account for nearly half of all STDs reported annually [1]. Further, increasing evidence indicates that infection with other STDs is significantly associated with HIV acquisition [2–4]. The increase in STD incidence requires new and innovative approaches to prevention.

Partner notification services (PNS) are among the most effective public health interventions to address transmission of STDs and are recommended by the Centers for Disease Control and Prevention (CDC) for all individuals presenting with HIV, infectious syphilis, or gonorrhea, as well as chlamydia if resources allow. As STDs often present with no symptoms, notification of possible exposure may be necessary to prompt screening among partners of individuals diagnosed with an STD [1,5]. In these situations, PNS is essential to interrupting the transmission of disease. The key personnel in PNS are disease intervention specialists (DIS), non-clinical personnel who interview index patients to identify these patients’ recent sexual partners and subsequently notify named partners of their potential exposure to an STD. Notification may take place by DIS referral (DIS notify partners directly) or contract referral (the index patient notifies their partner with the understanding that the DIS will reach out to partners who do not visit the health service by an agreed-upon date) [6]. PNS outcomes may be evaluated using several index- and partner-level outcomes including the time between STD diagnosis and index interview; number of partners named, tested, and treated; and time between index case diagnosis and partner treatment [7,8].

Significant challenges exist in implementation of successful PNS programs including barriers to engaging index cases and contacting partners, particularly given the prevalence of sex with anonymous partners [9,10]. Integrating DIS in clinical settings has been associated with reduced times to interview and increased number of partners named in an MSM patient population [10], and reduced times to interview and greater number of interviews completed, partners named, and partners treated in PNS for syphilis [11]. To improve PNS outcomes for syphilis and gonorrhea diagnoses in a general STD clinic population, we embedded DIS at the Rhode Island STD Clinic to conduct on-site PNS as a component of standard clinic care. The purpose of DIS integration was to overcome barriers to successful delivery of PNS by engaging index cases in person, thus more effectively facilitating notification of partners with a potential STD exposure. The primary goal of the present study was to evaluate the effectiveness of integrated DIS at an STD clinic in improving PNS outcomes, including the expediency of index patients interviewed and the number of partners named, tested, and treated.

## Materials and methods

### Setting

The Rhode Island STD Clinic is a walk-in clinic established at an outpatient hospital facility in January 2012 to address the increasing prevalence of STDs in Rhode Island and receives partial funding from the Rhode Island Department of Health (RIDOH). Rhode Island state law requires that the names of patients testing positive for infectious syphilis, gonorrhea, and chlamydia be reported to RIDOH for surveillance and partner notification purposes. In April 2015, DIS were embedded at the Rhode Island STD Clinic to help facilitate PNS on-site for infectious syphilis and gonorrhea. Prior to on-site integration, DIS were located mainly at the RIDOH and only contacted index cases following report of a new STD diagnosis. During the study period, PNS and clinic processes did not change and no new DIS were enlisted; existing DIS from the RIDOH were relocated to the STD Clinic. In Rhode Island, DIS complete a CDC training course in disease intervention methods prior to conducting PNS [12]. DIS utilize a standard interview guide formulated by the CDC in accordance with PNS delivery recommendations in order to optimize engagement of, and elicitation of partner information from, index patients [6,13].

Using a standard CDC interview guide [13], DIS conducted one-time interviews with patients at the time of testing if patients exhibited symptoms consistent with syphilis or gonorrhea, or when patients returned to the clinic to receive treatment for syphilis or gonorrhea. Patients provided verbal consent to participate in the interview, and participation was not required [6]. PNS is not routinely performed in Rhode Island for patients with chlamydia due to high incidence and limited resources. If DIS were unable to meet index cases in the clinic, DIS attempted to contact patients by phone, letter, field visit, or text message. DIS interviewed index cases and collected data on reasons for testing, signs and symptoms of infection, risk behaviors, and contact information for sexual and injection drug using partners. Depending on the referral method selected, the index patient or DIS then contacted partners to inform them of possible exposure to an STD and refer them to the Rhode Island STD Clinic for testing.

### Data collection

Review of patient data was approved by The Miriam Hospital Institutional Review Board (IRB), and all patient data were de-identified prior to analysis. The Miriam Hospital IRB waived the need for consent in this study. Data were reviewed for the eight months before implementation of embedded DIS at the STD clinic (August 2014 to March 2015) and for the eight months after implementation (May 2015 to December 2015), with a one-month implementation period to allow for wash-out of remaining activities by off-site DIS (April 2015). Data collected include STD diagnosis, patient demographic characteristics (age, sex, gender, race, ethnicity, insurance coverage), and sexual risk behaviors. Data reviewed also included whether DIS attempted to contact partners, whether contact attempts were successful, whether interviews were conducted, location of interviews, number of days between diagnosis and interview, and the number of partners reported, located, successfully contacted, tested, diagnosed with an STD, and treated.

### Data analysis

We calculated means and frequencies for demographic and behavioral variables and tested the relative distribution of variables between the two time periods using Chi-square, Fisher’s exact, and two-sided t-tests. Significance was defined as *p*<0.05. All statistical analyses were conducted using Stata/SE 13.1 (StataCorp, College Station, TX).

## Results

A total of 2,745 patient visits occurred at the Rhode Island STD Clinic during the study period; of these, 527 had a diagnosis of any STD (HIV, hepatitis C virus, gonorrhea, chlamydia, syphilis, or trichomonas), and 145 were diagnosed with gonorrhea or infectious syphilis and were referred for PNS (Table 1). The mean index patient age was 31.4 years (range: 17–68 years). The majority (94%) of index patients were male, 42% were non-white, and 20% were Hispanic or Latino/a. The most commonly reported (82%) sexual risk category was MSM (including men who have sex with both men and women), and over half (56%) of index patients were uninsured. Of the index patient sample 72% (*n* = 145) tested positive for gonorrhea and 33% tested positive for infectious syphilis, with eight index patients (6%) testing positive for both. Patients who presented to the clinic after on-site DIS implementation (*n* = 87) did not differ from those who presented before implementation (*n* = 58) on the basis of demographic or behavioral characteristics.

**Table 1:** Characteristics of index patients before and after implementation of an on-site DIS at an STD clinic.

	Before (n = 58)		After (n = 87)		Total (n = 145)		Chi-square *p*-value
	n	%	n	%	n	%	
**Age *(mean*, *SD)***	30.2	12.0	32.2	11.6	31.42	11.8	0.321^a^
**Gender**							
Male	56	97%	80	92%	136	94%	0.316^b^
Female	2	3%	7	8%	9	6%	
**Race**							
White	30	52%	54	62%	84	58%	0.261
Non-white	27	47%	33	38%	60	42%	
**Ethnicity**							
Hispanic or Latino/a	10	18%	19	22%	29	20%	0.530
Non-Hispanic or Latino/a	47	82%	68	78%	115	80%	
**Risk**							
MSM	47	82%	71	82%	118	82%	0.897
Non-MSM	10	18%	16	18%	26	18%	
**Health insurance status**							
Insured	32	55%	49	56%	81	56%	0.891
Uninsured	26	45%	38	44%	64	44%	
**HIV diagnosis, ever**	3	5%	3	3%	6	4%	0.683
**Anonymous sexual partner, past year**	34	59%	49	56%	83	57%	0.784
**Prior STD diagnosis, past year**	17	29%	20	23%	37	26%	0.392
**Newly identified gonorrhea diagnosis**	40	69%	65	75%	105	72%	0.448
**Newly identified syphilis diagnosis**	20	34%	28	32%	48	33%	0.773

^a^t-test,

^b^Fisher's exact test;

PNS: partner notification services, DIS: disease intervention specialist, STD: sexually transmitted disease, MSM: gay, bisexual, and other men who have sex with men

DIS attempted to contact all index patients, either in person in the clinic or by phone, during the study period. Prior to implementation of on-site DIS, 76% of index patients were interviewed; after implementation, the number of index patients interviewed increased significantly, to 92% (Table 2). Following implementation of on-site DIS, a significantly greater number of interviews were conducted in person at the STD clinic rather than by phone (64%, compared to 11% before, p<0.05). Further, a significantly greater number of those interviewed after on-site DIS implementation were interviewed on the day of diagnosis (85%, compared to 61% before, p<0.05).

**Table 2:** Partner notification outcomes before and after implementation of an on-site DIS at an STD clinic.

	**Before** (n = 58)		**After** (n = 87)		**Total** (n = 145)		**Chi-square** ***p*-value**
	***N***	***%***	***N***	***%***	***N***	***%***	
**Attempted to contact**	58	100%	87	100%	145	100%	-
**Interviewed**	44	76%	80	92%	124	86%	**0.007**
	***Among interviewed***						
	(n = 44)		(n = 80)		(n = 124)		
**Interview location**							
Clinic	5	11%	51	64%	56	45%	**<0.001**
Phone	39	89%	29	36%	68	55%	
**Interview on day of diagnosis**	27	61%	68	85%	95	77%	**0.003**
**Any partners named**	44	100%	74	93%	118	95%	0.163
**Any partners treated**	7	16%	17	21%	24	19%	0.408

DIS: disease intervention specialist, STD: sexually transmitted disease

There was no significant change in the number of index patients who named at least one partner (93% after implementation versus 100% before), nor in the number who had at least one partner treated (21% after versus 16% before). The average number of partners named (mean 3.36 partners after, 4.18 before; two-tailed *p* = 0.62) and treated (mean 0.25 after, 0.16 before; *p* = 0.30) also did not differ significantly between pre- and post-DIS integration periods.

## Discussion

Integration of DIS at a publicly funded STD clinic resulted in some improved PNS outcomes. Integrated DIS facilitated a higher rate of successful, same-day contact with index patients with positive gonorrhea and infectious syphilis, resulting in a significantly greater number of index patients interviewed, a greater proportion interviewed in person, and a greater proportion interviewed on the day of diagnosis. These findings indicate that integration of DIS at the point of testing and treatment addresses some barriers to index patient engagement. However, there was no significant difference in the number of sexual partners named, tested, or treated after DIS implementation.

Capturing index patients at the time of testing is a critical element of successful PNS delivery. In the present study, on-site DIS were able to interview patients on the day of diagnosis or treatment more frequently than when DIS were based off-site, which improved the likelihood of successfully engaging index patients. Our data showing greater success of on-site DIS interviewing index patients are consistent with prior studies, which have reported that integration of DIS or other public health officials into clinical settings resulted in a reduced time to interview and an increased number of index patients interviewed on the day of diagnosis [10,11].

Despite greater success engaging and interviewing index patients, there was no significant difference in the number of sexual partners named or treated with integration of on-site DIS. This suggests that factors outside of DIS engagement may pose barriers to successful PNS outcomes. The prevalence of sex with anonymous partners likely contributes to the low number of partners named, which remained consistently low for the duration of the study period. In studies of attitudes regarding partner notification among MSM, anonymous sexual encounters emerged as the most common explanation for failure to notify partners of STD diagnoses, with or without formal PNS [14,15]. In the present study, this may be attributed in part to prevalent use of smartphone applications used to meet sexual partners (“hookup apps”), particularly among MSM, as our prior research has revealed that 60% of MSM newly diagnosed with HIV in Rhode Island reported having met partners online in the past year [16]. Compared to meeting partners in person, using hookup apps to meet partners is associated with a higher number of anonymous sexual encounters, as well as increased risk for contracting gonorrhea and chlamydia [17]. Intimate partner violence (IPV) is another potential barrier to engagement in PNS. In a recent study at a publicly funded STD clinic, almost one-third of participants identified perceived risk of IPV as a barrier to partner notification [18]. Furthermore, stigma associated with sexual orientation [19] and discussion of sexual behaviors [20,21] may function as additional barriers to engagement in PNS.

Facilitators to successful PNS are also largely unknown. STD clinics may be particularly well suited to PNS activities given that patients have reported reduced stigma, increased confidentiality, and greater sensitivity to sexual minority experiences at STD clinics compared to general or primary care practices [22–24]. However, setting, characteristics of sexual partners, partnership dynamics, and additional factors that may impede or facilitate successful PNS delivery merit further study to inform improvements in PNS delivery, which are needed to improve identification and engagement of index patients’ partners. Potential avenues for future PNS implementation and research may include integration of hookup apps or other web-based platforms in DIS interviews and PNS outreach, as well as exploring the incorporation of patient-delivered partner notification programs.

This study is limited by its sample size and unique setting. The small sample size of index patients precludes the comparison of partner notification outcomes across variables such as gender, race, ethnicity or sexual behavior. Additionally, the current study was performed at one STD clinic, which may limit generalizability to PNS in other settings. Subsequent research should examine the effects of on-site DIS with a larger and more diverse patient population, and perhaps compare across different types of clinic settings. Another limitation to this study is recall bias, as patients were asked to retrospectively report partners to DIS. In the present study, patients who were referred to the clinic for testing through PNS were not linked to the index patients who identified them; however, future study analyzing partner referral networks may provide further insight into patient-level factors affecting successful PNS delivery.

## Conclusions

PNS is an integral part of STD prevention and a critical public health intervention. The present study demonstrates the improvement in engaging and interviewing index patients for PNS when DIS are integrated in an STD clinic setting. Future research should investigate other barriers to identifying partners of index patients and engaging them in STD testing through PNS. Additional methods of contacting partners for PNS such as email, social media, and through hookup apps in particular warrant future study.
